# Identification of two near-identical novel HIV-1 unique recombinant forms (CRF01_AE/B) among men who have sex with men in baoding, hebei, China

**DOI:** 10.3389/fgene.2023.1105739

**Published:** 2023-02-02

**Authors:** Binbin Zhang, Sisi Chen, Juan Meng, Miaomiao Su, Weiguang Fan, Weina An, Xinli Lu

**Affiliations:** ^1^ Laboratory of Endocrinology, Baoding No. 1 Central Hospital, Baoding, China; ^2^ Clinical Laboratory, The People’s Hospital of Baoding, Baoding, Hebei, China; ^3^ Infection Division, The People’s Hospital of Baoding, Baoding, Hebei, China; ^4^ Department of AIDS Research, Hebei Provincial Center for Disease Control and Prevention, Shijiazhuang, Hebei, China

**Keywords:** HIV, near full-length genome, unique recombination forms, baoding, MSM

## Abstract

Men who have sex with men (MSM) are the most frequent infection route of the human immunodeficiency virus (HIV) in Baoding, China, creating chances for the occurrence of unique recombinant forms (URFs) of the virus, i.e., recombination of different subtypes caused by co-circulation of multiple subtypes. In this report, two near-identical URFs (BDD002A and BDD069A) isolated from MSM in Baoding were identified. Phylogenetic tree analysis based on nearly full-length genomes (NFLGs) revealed that the two URFs formed a distinct monophyletic cluster with a bootstrap value of 100%. Recombinant breakpoints analysis identified that the NFLGs of BDD002A and BDD069A were both composed of CRF01_AE and subtype B, with six subtype B mosaic segments inserted into the CRF01_AE backbone. The CRF01_AE segments of the URFs clustered closely with the CRF01_AE reference sequences, and the B subregions clustered with the B reference sequences. The recombinant breakpoints of the two URFs were almost identical. These results suggest that effective interventions are urgently needed to prevent the formation of complex HIV-1 recombinant forms in Baoding, China.

## Introduction

The human immunodeficiency virus-1 (HIV-1) possesses an extremely high mutation frequency, resulting in HIV-1-enriched gene polymorphisms (Yebra et al., 2018). During the spread of HIV, if more than two subtypes infect the same cell, their genomic information can exchange to generate recombinant virus genomes (Moore and Hu, 2009). A total of 132 circulating recombinant forms (CRFs) of HIV and many unique recombinant forms (URFs) have been reported worldwide (https://www.hiv.lanl.gov/content/sequence/HIV/CRFs/crfs). In recent years, CRF01_AE and subtype B have become the two main HIV-1 genotypes prevalent in key populations of China, especially among men who have sex with men (MSM) (He et al., 2012). Hebei is a northern province of China with low HIV prevalence (Lu et al., 2017). By the end of October 2020, 15,178 individuals in the Hebei Province were diagnosed with HIV-1/AIDS, and the number of individuals infected with HIV-1 through MSM reached 77.5% (Lu et al., 2020; Wang, 2020). Baoding, which borders Beijing and Tianjin, was the second region severely affected by HIV-1 in Hebei Province, with MSM responsible for 84.8% of the HIV-1-infected population (Shi et al., 2021a). The prevalence of subtypes CRF01AE and B was similar to that reported in the whole province at 49.44% and 17.78%, respectively (Shi et al., 2021b). In this study, we identified two highly similar, nearly full-length genome (NFLG) sequences isolated from MSM in Baoding, Hebei Province. These two unique recombinant forms (URFs) were composed of subtypes CRF01_AE and B.

## Case description

The two individuals, BDD002A and BDD069A, were a 35-year-old unmarried man and a 39-year-old married man, respectively. Moreover, their baseline CD4^+^ T-cell counts were 242 cells/ul and 128 cells/ul, respectively while the HIV-1 viral load were 1,140,000 copies/ml and 703,000copies/ml, respectively. They were infected through homosexual transmission. The study was approved by the Medical Ethics Committee of the Baoding People’s Hospital (protocol number: 2019-03). Written informed consent was obtained from the subjects prior to sample collection.

## Diagnostic assessment

As described previously by us (Yang et al., 2022), HIV-1 RNA was extracted from 140 µL of the plasma samples of subjects BDD002A and BDD069A using a QIAamp Viral RNA Mini Accessory Set (QIAGEN, Hilden, Germany). PrimeScript IV 1st Strand cDNA Synthesis Mix (TaKaRa Biotechnology, Dalian, China) was used to reverse transcribe the RNA into 3′half-molecule cDNAs using the primers and reaction conditions listed in our previous report (Yang et al., 2022). Nested polymerase chain reactions (PCR) was performed using TaKaRa Premix Taq (TaKaRa Biotechnology, Dalian, China) to amplify 3′halfmolecule region of the NFLGs of BDD002A and BDD069A. HIV-1 near full-length *pol* and *gag* genes were amplified using Takara One-step RT-PCR Kit v2.0 (TaKaRa Biotechnology, Dalian, China). HIV-1 near full-length *pol* gene was amplified in two steps using the primers below:Pol-1e: TGGAAA TGTGGRAARGARGGAC (forward), Pol-x: CCTGTAATG CARMCCCCAATATG TT (reverse) and Pol-3: ACT​GAG​AGA​C AG​GCT​AAT​TTT​TTA​GGG​A (forward), Pol-4e:CTCCTAGTGG GGATRTGTACTTCTGARCTTA (reverse). HIV-1 near full-length *gag* gene was amplified in two steps using the primers below:gag-763:TGACTAGCGGAGGCTAGAAGG (forward), gag-5:TTCCYCC TATCATT TTTGGTTTCC (reverse) and gag-617: TGT​GGA​AAA​T CT​CTA​GCA​GTG​G (forward), gag-6:TAATGCTTTTATT TTYTCYT CTGTCAATGGC (reverse). The reaction conditions used for amplification have been reported previously (Lu et al., 2017; Fan et al., 2022a). The positive PCR products were purified using 1.0% agarose gel electrophoresis and sequenced using the Sanger sequencing technology by Tianyi Huiyuan Bioscience & Technology Inc. (Beijing, China). Two NFLGs were obtained by assembling them with near full-length *gag*, *pol* and 3′half-genome gene sequences using Sequencher 5.4.6 (Gene Codes Corp., Ann Arbor, MI, United States of America).

The NFLG sequences were then submitted to the online tool HIV BLAST (https://www.hiv.lanl.gov/content/sequence/BASIC_BLAST/basic_blast.html) to determine whether the same recombinant sequences had been identified previously, but no sequences with high similarity (>95%) to BDD002A and BDD069A were found in the HIV database. In addition, phylogenetic tree and subregion phylogenetic trees were constructed using the neighbor-joining (N-J) method based on the Kimura two-parameter model with 1,000 bootstrap replications by Mega6.0. The recombination pattern was determined by Recombination Identification Program (https://www.hiv.lanl.gov/content/sequence/RIP/RIP.html) and jpHMM. Recombination breakpoints were identified by SimPlot (v3.5.1.0) and Bootscan analysis.

**FIGURE 1:**
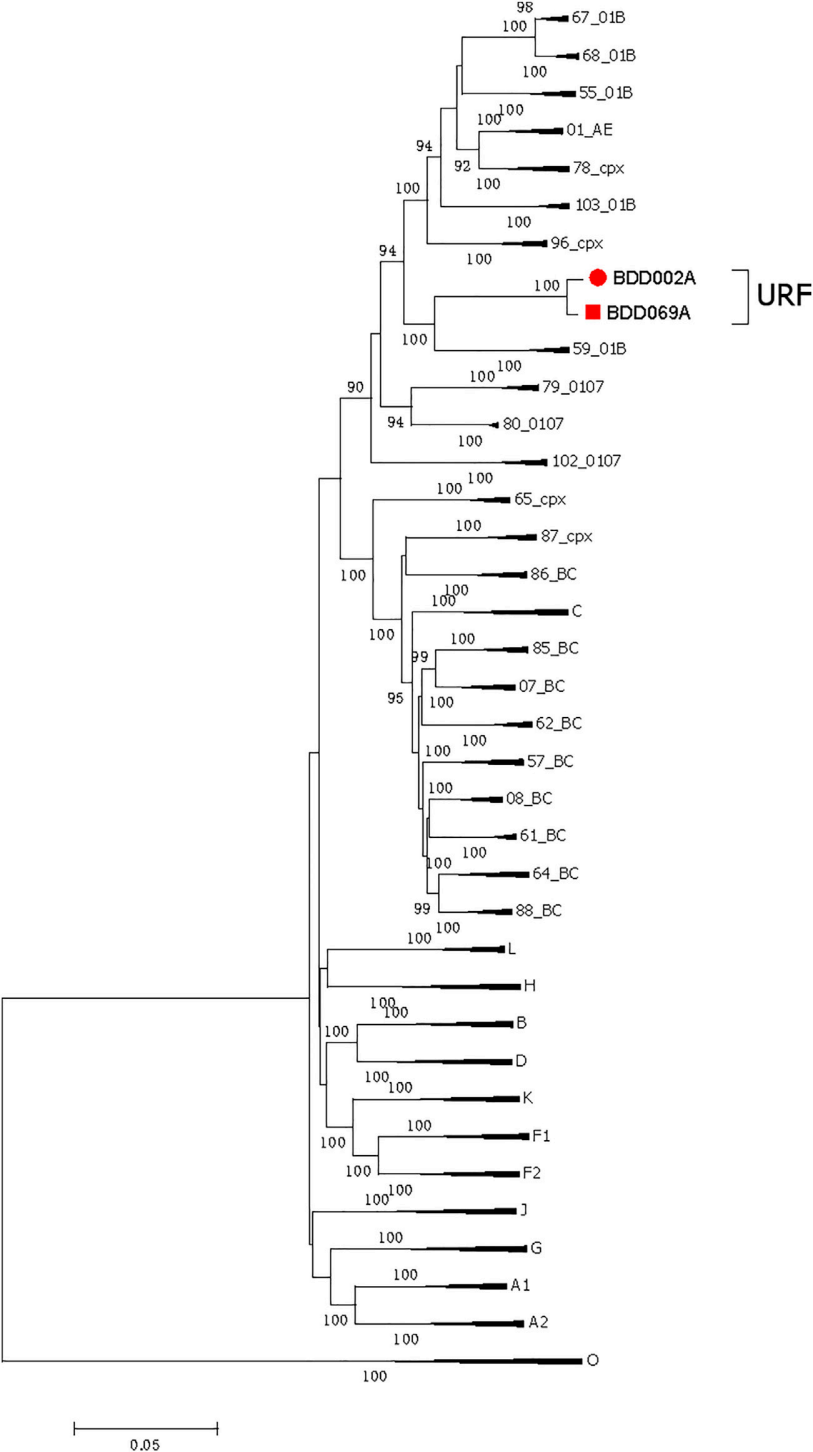
**T**he phylogenetic tree is based on NFLG sequences BDD002A and BDD069A. The standard subtype references were downloaded from the Los Alamos National Laboratory HIV Database (www.hiv.lanl.gov). The neighbor-joining phylogenetic tree of BDD002A (8,810 bp, red-filled circle●) and BDD069A (8,944 bp, red-filled square■) was constructed based on the NFLG sequences using Mega6.0. The stability of each node was assessed by bootstrap tests with 1,000 replicates, and only bootstrap values ≥90% are shown at the corresponding nodes. The scale bar represents a 5% genetic distance. NFLG, near full-length genome.

**FIGURE 2:**
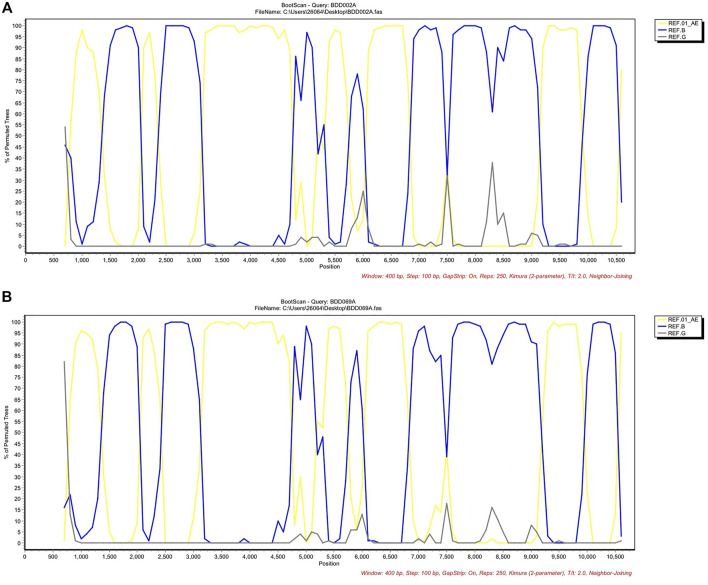
Bootscan results of the novel CRF01_AE/B identified. **(A)** Bootscan plots of BDD002A using CRF01_AE, subtype B and subtype G as references. The parameters of the bootscan analysis were a window size of 400 bp and a step size of 100 bp. **(B)** Bootscan plots of BDD069A using CRF01_AE and subtype B as putative parental reference sequences and subtype G as the outgroup. The parameters of the bootscan analysis were a window size of 400 bp and a step size of 100 bp. CRF, circulating recombinant form.

**FIGURE 3:**
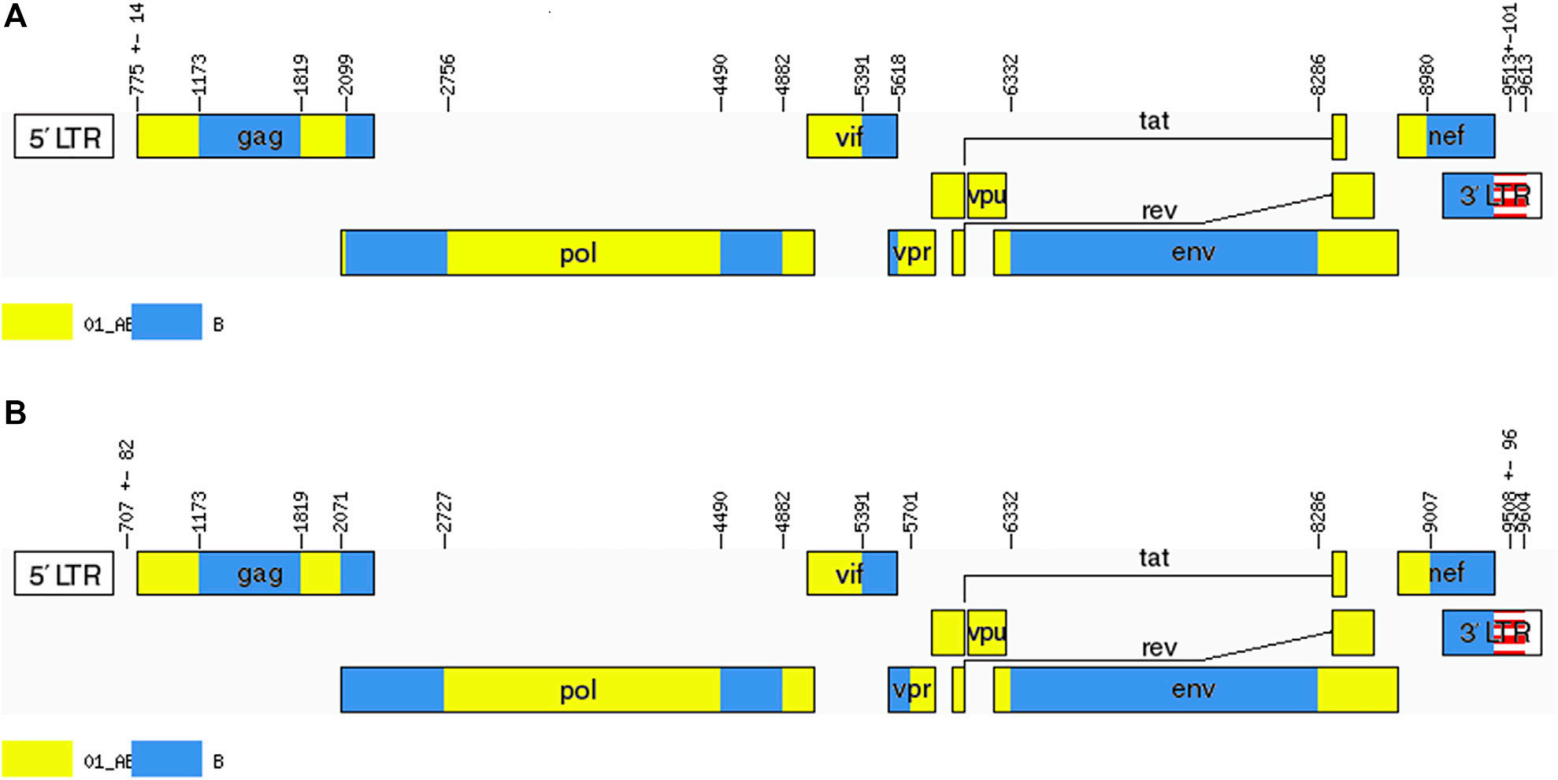
Recombinant maps of the novel CRF01_AE/B identified. The unique recombinant maps of **(A)** BDD002A and **(B)** BDD069A were drawn with the online Recombinant HIV-1 Drawing Tool (https://www.hiv.lanl.gov/content/sequence/DRAW_CRF/recom_mapper.html). Six B segments were inserted into the CRF01_AE backbone, and 11 breakpoints divided the whole genome into 12 unique segments.

**FIGURE 4:**
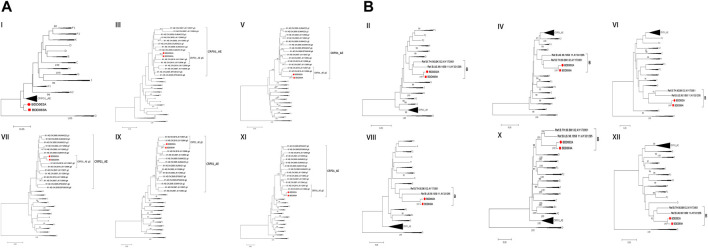
Subregional phylogenetic trees of the novel CRF01_AE/B identified. The trees were constructed by the neighbor-joining method with 1,000 bootstrap replications using Mega6.0. Bootstrap values ≥90% are shown at the corresponding nodes. The scale bars indicate a genetic distance of 5%. Each segment of BDD002A and BDD069A is marked by a red-filled circle and a red-filled square, respectively. **(A)** CRF01_AE regions for both URFs. **(B)** subtype B regions for both URFs.

We acquired two NFLG sequences with 8,810 bp (HXB2: 761–9,613) and 8,944 bp (HXB2: 625–9,604) from BDD002A and BDD069A, respectively. The constructed NFLG N-J tree showed that both BDD002A and BDD069A formed a monophyletic branch with a bootstrap value of 100%, indicating that BDD002A and BDD069A are two novel recombinant forms (Figure 1). The recombinant breakpoints analysis revealed that BDD002A and BDD069A were composed of 12 interlaced mosaic gene segments, including six CRF01_AE subregions (I, III, V, VII, IX and XI) and 6 B regions (II, IV, VI, VIII, X and XII), with 11 recombinant breakpoints relative to the HXB2 coordinate (Figure 2; Figure 3; Figure 4). Subregion analysis (Figure 3) confirmed that the gene mosaic structure of the two NFLGs are: ICRF01_AE (HXB2, 790–1,172 nt), IIB (HXB2, 1,173–1,818 nt), IIICRF01_AE (HXB2, 1,819–2,098 nt), IVB (HXB2, 2,099–2,755 nt), VCRF01_AE (HXB2, 2,756–4,489 nt), VIB (HXB2, 4,490–4,881 nt), VIICRF01_AE (HXB2, 4,882–5,390 nt), VIIIB (HXB2, 5,391–5,617 nt), IXCRF01_AE (HXB2, 5,618–6,331 nt), XB (HXB2, 6,332–8,285 nt), XICRF01_AE (HXB2, 8,286–8,979 nt) and XIIB (HXB2, 8,980–9,411 nt) for BDD002A (Figure 3A); and ICRF01_AE (HXB2, 790–1,172 nt), IIB (HXB2, 1,173–1,818 nt), IIICRF01_AE (HXB2, 1,819–2,070 nt), IVB (HXB2, 2,071–2,726 nt), VCRF01_AE (HXB2, 2,727–4,489 nt), VIB (HXB2, 4,490–4,881 nt), VIICRF01_AE (HXB2, 4,882–5,390 nt), VIIIB (HXB2, 5,391–5,700 nt), IXCRF01_AE (HXB2, 5,701–6,331 nt), XB (HXB2, 6,332–8,285 nt), XI CRF01_AE (HXB2, 8,286–9,006 nt) and XIIB (HXB2, 9,007–9,411 nt) for BDD069A (Figure 3B). The above data revealed that both NFLGs shared seven almost identical breakpoints except a minor difference within the *vif-vpr* gene region (Figure 3). The parental origin of all fragments of the two NFLGs were analyzed and the CRF01_AE regions for both URFs were from the CRF01_AE cluster five lineage, which is circulating primarily among MSM in major northern cities of China (Figure 4A) (Feng et al., 2013). The subtype B regions for both URFs were clustered within the northern China subtype B lineage, which also circulates primarily among MSM in northern China (Figure 4B) (Li et al., 2011).

## Discussion

The diversity of HIV-1 is a significant challenge in preventing the global spread of HIV due to the different pathogenicity of subtypes. The co-circulation of multiple subtypes enables the occurrence of numerous URFs (Gao et al., 2021). In China, the prevalence of CRFs may be underestimated because of the high prevalence of URFs and the formation of many potential CRFs (Liu et al., 2019; Wang et al., 2021). URFs convert to CRFs if they spread widely and circulate in the population (Robertson et al., 2000; Hemelaar, 2012). In recent years, new CRFs such as CRF103_01 B and CRF112_01 B have been found because of the high occurrence of URFs among MSMs in Baoding (Zhou et al., 2020; Fan et al., 2022b; Wang et al., 2022). The continuous emergence of new recombinant forms has brought new barriers to the monitoring, treatment, vaccine development and prevention of HIV. In this study, we identified and characterized two novel recombinants derived from subtypes CRF01_AE and B, which were highly different from those reported previously in the cities of Langfang and Baoding and other Chinese provinces (Guo et al., 2014; Yan et al., 2015; Li et al., 2018; Huang et al., 2019; Li et al., 2019; Ou et al., 2019; Ge et al., 2020; Fan et al., 2022c). These two URFs were more complex than those reported previously. Although no new CRF has been formed, we infer that it may be a potential CRF. Currently, the sexual contact has been the main route of HIV-1 spread, it is key vital for us to carry out dynamic monitoring of new recombination forms in order to interdict HIV-1 spread.

In conclusion, we identified two almost identical HIV-1 URFs among MSMs in Baoding. The recombinant forms of CRF01_AE/B and CRF01_AE/CRF07_BC were found frequently in the MSM population of Baoding, indicating an increase in the genetic diversity of HIV-1 and the presence of many undiagnosed multiple infection cases in this region (Ou et al., 2019; Fan et al., 2022d; Yang et al., 2022), further bringing more barriers for HIV prevention and therapy. This study suggests that we should continuously monitor HIV-1 molecular epidemiology in order to provide effective suggestions to HIV-1 prevention and vaccine design.

## Data Availability

The datasets presented in this study can be found in online repositories. The names of the repository/repositories and accession number(s) can be found below: https://www.ncbi.nlm.nih.gov/genbank/, OP796643 https://www.ncbi.nlm.nih.gov/genbank/, OP796644.
